# Supplementary material for: Globally asynchronous sulphur isotope signals require re-definition of the Great Oxidation Event
Source: Nat Commun. 2018 Jun 8;9:2245. doi: 10.1038/s41467-018-04621-x (PMC5993798; doi:10.1038/s41467-018-04621-x)
Supplement: Supplementary file 1 — Supplementary Information [file 41467_2018_4621_MOESM1_ESM.pdf]

SUPPLEMENTARY INFORMATION

Globally asynchronous sulphur isotope signals require re-definition of the Great Oxidation Event

Pascal Philippot[1,2*], Janaína Ávila[3], Bryan Killingsworth[4], Svetlana Tessalina[5], Franck Baton[1], Tom Caquineau[1], Elodie Muller[1], Ernesto Pecoits[1,**], Pierre Cartigny[1], Stefan Lalonde[4], Trevor Ireland[3], Christophe Thomazo[6], Martin van Kranendonk[7], Vincent Busigny[1]

Supplementary Note 1 - Geological setting

The Turee Creek group stratigraphy has been described as a broad shallowing-upward profile from deep-water banded iron formation of the Boolgeeda Iron Formation, through fine-grained siliciclastic deposits of the Kungarra Formation, to fluvial and shallow marine strata of the Koolbye and Kazput formations[1,2]. Diamictites of the Meteorite Bore Member (MBM) and a second[3] or possibly third[4] recently discovered units of glacial diamictites were interpreted to be deposited from a floating ice sheet over a marine basin[5]. Sedimentation of the Turee Creek Group is considered to have occurred in an asymmetric basin, called the McGrath Trough, which reflects flexural subsidence driven by the migration to the northeast of a geanticline or a thrust-fold belt system[1,5]. More recently, Van Kranendonk et al. (2015)[6] proposed that the Turee Creek Group was deposited in an intracratonic basin that deepened to the northwest, with terrigenous input sourced from erosion of uplifted bedrock in the southeast.

The chronology of the Turee Creek Group and broad correlation to timing of the Great Oxidation Event and to other Paleoproterozoic basins is constrained by: (i) detailed stratigraphic reconstruction from the Hardey Syncline (~4,000 m-thick), Deepdale (~2,500 m thick) and Boundary Ridge (7 m-thick) sedimentary successions (Supplementary Fig. 1); (ii) three U-Pb zircon ages of 2,450 ± 3 Ma[7], 2,340 ± 22 Ma[8] and 2,209 ± 15 Ma[2] at the base of the underlying Boolgeeda Iron Formation (Woongarra rhyolites), at the base of the Meteorite Bore Member diamicites, and at the base of the Wyloo Group (Cheela Springs Basalt), respectively; and (iii) sulphur-isotope data obtained on the Boundary Ridge area[9,10] located about 50 km north west of the Hardey Syncline. The occurrence of thin (1 to 3 meters thick) glaciogenic diamictites at the Boundary Ridge and Deepdale sections are significant in terms of interpreted lithostratigraphy. Martin et al. (1999)[1] interpreted the glaciogenic diamictites at Deepdale to be part of the Boolgeeda BIFs, and therefore that the glacial diamictites were deposited as part of the Boolgeeda Iron Formation in a deep water part of the basin. In contrast, Van Kranendonk (2010)[11] and Van Kranendonk et al. (2015)[6], argued that the glaciogenic horizon at the Boundary Ridge was located at the transition between the Boolgeeda BIF and overlying Kungarra

Formation and therefore that it could be correlated with the 400 m-thick Meteorite Bore Member 50 km to the south-east. Williford et al., (2013)[9], using this stratigraphic interpretation in combination with sulphur isotope data obtained at the Boundary Ridge, suggested that the Boundary Ridge and Meteorite Bore Member glaciogenic diamictites were deposited at the same time during the final stage of the Great Oxidation Event. In contrast, Swanner et al., (2013)[10], using a different set of sulphur isotope data obtained at the Boundary Ridge, proposed that the Boundary Ridge diamictite was a temporally distinct, older, unit relative to the Meteorite Bore Member, which is in agreement with the stratigraphic interpretation of Martin et al (1999)[1].

The 2 m-thick diamictite horizon located within the Boolgeeda Iron Formation at about 20 meters below the contact with the overlying Kungarra Formation of our T1 drill core (Supplementary Fig. 2 d,e,f) shows the same textural characteristics as the one identified at the Boundary Ridge locality (Supplementary Fig. 2 g,h). The evidence comes from several ~5 cm pebbles and several cm-scale pebbles isolated in a siltstone matrix in a meter-scale layer. This clearly demonstrates that the Boundary Ridge diamictite represents a distinct glacial event separated from the overlying MBM by nearly 1500 metres of the Kungarra shales. Our new Re-Os sulphide age of ~2.31 Ga (Supplementary Fig. 9) for the base of the MBM supports this interpretation, namely that the deep basin glaciomarine horizon identified in the Boolgeeda IF is much older and most likely correlative with the first Huronion glacial event (Ramsay Lake) at about 2.45 Ga. This interpretation is further confirmed by a new U-Pb age of 2.454 ± 23 Ma obtained on detrital zircons extracted from the diamictite horizons of the Boundary Ridge and T1 core (Hardey Syncline)[9].

Supplementary Note 2 - Drilling and samples

Drilling was performed in the Hardey Syncline area, which represents the most complete exposures of the Turee Creek Group (Supplementary Fig. 1a). Geological mapping of surface outcrops was undertaken in order to best assess where to locate the drill sites, in reasonably accessible areas, away from faulting, and where representative stratigraphic sections were preserved. Several geological sections were made through the syncline at various places along strike. Three drill sites were chosen in the northwestern (T1), and southern (T2 and T3) parts of the Hardey Syncline (Supplementary Fig. 1b). The first hole (T1, 22°48'31.00"S-116°47'15.90"E) was drilled with RC hammer to a depth of 98.8 m and diamond drilling with NQ core (47.6 mm diameter) from 98.8.0 to 130.9 m through the base of the Kungarra Formation and from 130.9 to 174.9 m through the top of Boolgeeda Iron Formation. The second hole (T2, 22°50'49.70"S - 116°52'27.70"E) intercepted the base of the Meteorite Bore Member diamictites. Drilling was performed with RC hammer to a depth of 117.7 m and diamond drilling with NQ core from 117.7 to 281.7 m through the MBM diamictites and from 281.7 to 384.9 m through the underlying mudstone, siltstone, sandstone and carbonate stromatolite of

the Kungara Formation. The third hole (T3, 22°52'15.90"S - 116°56'46.40"E) was drilled with RC hammer to a depth of 75.8 m and diamond drilling with NQ core from 75.8 to 182.4 m through the base of the Kazput Formation and from 182.4 to 189.3 m through the top of Koolbye Formation quartzites. The three drillcores were analysed for their major and trace element compositions using X-ray core scanning at high stratigraphic resolution (2 cm step). This compositional dataset has permitted robust localisation of sedimentological contacts, both sharp and transitional as well as critical zones of elemental enrichments, revealing shifts in clastic input (Al, Ti, Zr) and enrichment of redox-sensitive elements (S, Fe, Mn, Cr) (work in preparation). The main lithologies exposed at the surface are shown in Supplementary Fig. 3 together with their drill core equivalent collected at depths. The 135 samples from the three drill cores were collected to reconstruct a chemostratigraphic record of sulphur isotopes throughout the Turee Creek Group. In addition, 5 samples were selected at the base of the Meteorite Bore Member diamictites to perform Re-Os chronology.

The Meteorite Bore Member represents a massive package of ~400 m thick glacial diamictite showing no apparent bedding. The sequence is composed of chlorite-bearing mudstone containing randomly distributed dropstones of various sizes (mm- to m-scale) and origin (carbonate, chert, gneiss, quartzite and local pockets of sandstone showing convolute structure). The samples used for Re-Os dating were collected throughout a ~20 meter scale interval between 252.55 and 272.46 metres depth. This section consists of a uniform mudstone matrix with local dropstones one to several centimetres in size (Supplementary Figure 3d, sample T2-253.3) and randomly distributed nodular aggregates of pyrites interpreted to be of early diagenetic origin (see below). For the five diamictites used for bulk rock dating (T2-252.55, T2-259.3, T2-264.3, T2-271.1 and T2-272.46, Supplementary Table 1), we were careful to only collect the mudstone part of the sample and avoid portions with dropstones. The reasons for choosing the Meteorite Bore Member diamictite for dating are as follows. First, bulk-rock chemical analyses showed that the entire section is relatively static in composition with sulphur and Total Organic Content (TOC) contents of about 1300 to 2300 ppm and 500 to 1000 ppm, respectively. Second, a detrital zircon U-Pb age of 2340 ± 22 Ma obtained by Caquineau et al (2018)[8] was obtained in the same sedimentary interval, thus providing an independent test of evaluating the significance of the Re-Os age. Third, a detrital zircon U-Pb age of 2.454 ± 23 Ma was obtained by Caquineau et al (2018)[8] on the diamictite horizon of the Boolgeeda Iron Formation at the base of the T1 core (Figure 3). Since the Boolgeeda Iron Formation in T1 and the overlying Kungarra Formation containing the MBM diamictites in T2 are in sedimentary continuity, this age constraint offered a second independent mean for evaluating the significance of the Re-Os age as well as the rate of sedimentation of the Kungarra Formation.

Supplementary Note 3 - Textures and chemical composition of sulphides

In all samples, preliminary identification of sulphides was performed by standard petrographic analyses of thin sections (Supplementary Figs. 4, 5, 6). Distinction between syngenetic/diagenetic pyrites, and detrital and epigenetic pyrites was based on sulphide chemistry, host lithology, and textural features.

Host lithologies that may contain detrital sulphides identified from their rounded appearance include the Koolbye Formation quartzites at the base of T3 (Figs. 2 and 4) and the 5-meter thick sandstone bed located beneath the Meteorite Bore Member diamictites (base of T2; Figs. 2 and 5). Only a few sulphides were analysed in these rocks and special care was taken to discard rounded grains of potential detrital origin, as these are not expected to preserve information on the sulphur cycle in the depositional environment of the host rock. Similarly, none of the sulphides present in dropstones of the Meteorite Bore Member and of the Boolgeeda Iron Formation (Supplementary Figs. 2, 3) were analysed either by *in situ* or bulk rock techniques.

Detailed petrographic analyses indicate that the majority of the pyrites studied here show textures indicative of a syngenetic or early diagenetic origin. Examples of such textures include rounded and elongated zoned nodules, concretions, microcrystalline aggregates, framboidal cores, finely disseminated pyrite (<10 μm), small euhedral and subhedral crystals (< 25 μm) aligned with bedding (or conform to soft sediment deformation structures), and bands of closely spaced fined grained pyrite (Supplementary Figures 4, 5, 6). The relative arrangement and proportion of these different textural types in a particular horizon is variable but all forms are interpreted to originate during early diagenesis or, in the case of pyrite framboids, possibly syngenetically within the water column or diagenetically below the sediment-water interface[13]. A few samples, however, show textures indicative of a later generation of diagenetic pyrite. These include overgrowth of coarser euhedral to subhedral pyrites as well as individual large euhedral to subhedral pyrite crystals (> 25 μm). Pyrite overgrowths are regarded as diagenetic rather than from metamorphic and/or hydrothermal events as they occur associated with poorly permeable lithologies (e.g., mudstone) with no evidence of pyrite recrystallisation along schistosity planes. Coarser textures and overgrowth zones (see below) are interpreted to be the result of local re-mobilisation of Fe and S from smaller earlier pyrite generations. Of all samples studied, only one (T1-169.90) is known to record epigenetic pyrite as indicated by the presence of large, inclusion-free, euhedral pyrite crystals in a mm-scale vein.

Sulphur isotopic signatures provide another line of evidence for a sedimentary (syngenetic/diagenetic) origin of the pyrites. A syngenetic origin is particularly clear for pyrites in the 1.5 m-thick green siltstone horizon of the Boolgeeda IF, showing strong enrichment in sulphur content (up to 5 weight %), together with strongly negative $\delta^{34}S$ values (down to -35‰) and $\Delta^{33}S$ = 0‰ (Fig. 3). In these samples, sulphide occurs in association with quartz forming millimetre-scale layers parallel to the bedding in a matrix mainly composed of Fe-chlorite, Fe-oxides (magnetite and hematite) and apatite (Supplementary Figs. 5c, d and 6). Such layering cannot be formed by secondary processes, but instead reflects periodic delivery of non-anomalous sulphur component to the water column. Finally,

authigenic sulphides that incorporate sulphur from the environment of deposition can display ranges of $\delta^{34}S$ and $\Delta^{33}S$ values, which are indicative of different sources and processes (photolitic, biological and/or nonbiological redox fractionation reactions at the time of deposition).

Further support for the syngenetic/diagenetic nature of sulphides is given by inter-element ratios, mostly Co/Ni but also Cu/Ni and Zn/Ni, determined by LA-ICP-MS. Previous studies[14-17] have shown that Co/Ni ratio is an effective chemical indicator for the environment of pyrite formation. Volcanogenic-hydrothermal pyrite is generally characterized by a high Co content with a high Co/Ni ratio[18-20]. On the other hand, high Ni and Co/Ni ≤ 2 have been observed in pyrite that forms in organic matter-rich environments[21,22]. Large et al. (2014)[16] used Co/Ni ratios as a chemical screening for sedimentary pyrites. On a Co versus Ni plot, samples which showed a linear array parallel to Co/Ni = 1 and with Co/Ni < 2 were selected as suitable indicators of a diagenetic origin[16]. In a recent study, Gregory et al., (2015)[17] have defined several inter-element ratios that can be used as chemical proxies for sedimentary pyrites. Characteristic values and composition limits of sedimentary pyrites were determined to be: 0.01 < Co/Ni <2, 0.01 < Cu/Ni < 10, 0.01 < Zn/Ni < 10, 0.1 < As/Ni < 10, Ag/Au>2, 1 < Te/Au < 1000, Bi/Au > 1, Sb/Au > 100, and As/Au >200.

Here, we applied to our dataset only the Ni-based ratios as discriminants as they are more robust indicators due to their generally high abundance in pyrite. In addition, Co and Ni have similar ionic radii to Fe and tend to be incorporated into the structure of pyrite. The behaviour of other elements, like As and Au, is more complex due to their mobility over a wide range of redox conditions. Forty-two (42) samples have been selected for trace element analysis based on pyrite texture, lithology, and stratigraphic unit. The Co/Ni ratios measured show that the majority of the samples (40 out of 42) have Co/Ni ≤ 2 within errors and all samples have Cu/Ni and Zn/Ni < 10 (Supplementary data 1, Supplementary Figs. 7 and 8). Some samples show very low trace element abundances, which can be explained by the low trace element abundance observed in the host rocks. Several studies have highlighted that the trace element composition of sedimentary pyrites is highly dependent on the water chemistry as such the trace element abundance variations observed along the stratigraphic profiles (Supplementary Fig. 7) could be explained by changes on water chemistry and redox conditions (this aspect will be discussed in a separate paper). Variations of trace element composition do not show correlation with the different textural pyrite types described previously.

Supplementary References

1. Martin, D. Depositional setting and implications of Paleoproterozoic glaciomarine sedimentation in the Hamersley Province, Western Australia. GSA Bulletin 111, 189-203 (1999).
2. Martin, D., Li, Z. X., Nemchin, A. A. & Powell, C. A pre-2.2 Ga age for giant hematite ores of the Hamersley Province, Australia. Econ. Geol. 93 (1998).
3. Van Kranendonk, M. & Mazumder, R. Two Paleoproterozoic glacio-eustatic cycles in the Turee Creek Group, Western Australia. Geol. Soc. Am. Bull. 127, 596–607 (2013).

4. Krapez, B., Müller, S. G., Fletcher, I. R. & Rasmussen, B. A tale of two basins? Stratigraphy and detrital zircon provenance of the Palaeoproterozoic Turee Creek and Horseshoe basins of Western Australia. Precamb. Res. 294, 67-90 (2017).
5. Martin, D. M., Powell, C. M. & George, A. Stratigraphic architecture and evolution of the early Paleoproterozoic McGrath Trough, Western Australia. Precambrian Research 99, 33-64 (2000).
6. Van Kranendonk, M. J., Mazumder, R., Yamaguchi, K. E., Yamada, K. & Ikehara, M. Sedimentology of the Paleoproterozoic Kungarra Formation, Turee Creek Group, Western Australia: A conformable record of the transition from early to modern Earth. Precamb. Res. 256, 314-343 (2015).
7. Trendall, A. F., Compston, W., Nelson, D. R., de Laeter, J. R. & Bennet, V. C. SHRIMP zircon ages constraining the depositional chronology of the Hamersley Group, Western Australia. Aust. J. Earth Sci. 51, 621-644 (2004).
8. Caquineau, T., Paquette, J.-L. & Philippot, P. U-Pb detrital zircon geochronology of the Turee Creek Group, Hamersley Basin, Western Australia: timing and correlation of the Paleoproterozoic glaciations. Precamb. Res. 307, 34-50 (2018).
9. Williford, K. H., Van Kranendonk, M. J., Takayuki, U., Reinhard, K. & Valley, J. W. Constraining atmospheric oxygen and seawater sulfate concentrations during Paleoproterozoic glaciation: In situ sulfur three-isotope microanalysis of pyrite from the Turee Creek Group, Western Australia. Geochim. Cosmochim. Acta 75, 5686–5705 (2011).
10. Swanner, E. D. et al. Geochemistry of pyrite from diamictites of the Boolgeeda Iron Formation, Western Australia with implications for the GOE and Paleoproterozoic ice ages. Chem. Geol. 362, 131-142 (2013).
11. Van Kranendonk, M. J. Three and a half billion years of life on Earth: a transect back into deep time. Vol. Record 2010/21 93pp. (Geol. Surv. West. Australia, 2010).
12. Ludwig, K. Isoplot/Ex, version 2.2: a geochronological toolkit for Microsoft Excel, Berkeley Geochron. Cent. Spec. Pub. 1a 46 (2000).
13. Wilkin, R. T., Barnes, H. L. & Brantley, S. L. The size distribution of framboidal pyrite in modern sediments: An indicator of redox conditions. . Geochim. Cosmochim. Acta 60, 3897-3912 (1996).
14. Loftus-Hills, G. & Solomon, M. Cobalt, nickel and selenium in sulphides as indicators of ore genesis. Mineral. Deposita 2, 228-242 (1967).
15. Bajwah, Z. U., Seccombe, P. K. & Offler, R. Trace element distribution, Co: Ni ratios and genesis of the Big Cadia iron-copper deposit, New South Wales, Australia. Mineral. Deposita 22, 292-300 (1987).
16. Large, R. R. et al. Trace element content of sedimentary pyrite as a new proxy for deep-time ocean–atmosphere evolution. Earth and Planetary Science Letters 389, 209-220 (2014).
17. Gregory, D. D. et al. Trace element content of sedimentary pyrite in black shales. Econ. Geol. 110, 1389-1410 (2015).
18. Hawley, J. E. & Nichol, I. Trace elements in pyrite, pyrrhotite and chalcopyrite of different ores. Econ. Geol. 56, 467-487 (1961).
19. Roscoe, S. M. Geochemical and isotopic studies, Noranda and Matagami areas. Can. Inst. Min. Metall. Trans. 65, 279-285 (1965).
20. Kimberley, M. M., Tanaka, R. T. & Farr, M. R. Composition of middle Precambrian uraniferous conglomerate in the Elliot Lake-Agnes Lake area of Canada. Precamb. Res. 12, 375-392 (1980).
21. Dill, H. & Kemper, E. Crystallographic and chemical variations during pyritization in the upper Barremian and lower Aptian dark claystones from the Lower Saxonian Basin (NW Germany). Sedimentology 37, 427-443 (1990).
22. Guy, B. M., Beukes, N. J. & Gutzmer, J. Paleoenvironmental controls on the texture and chemical composition of pyrite from non-conglomeratic sedimentary rocks of the Mesoarchean Witwatersrand Supergroup, South Africa. South African J. Sci. 113, 195–228 (2010).
23. Kendall, B., Creaser, R.A., Calver, C.R., Raub, T.D., Evans, D.A.D. Correlation of Sturtian diamictite successions in southern Australia and northwestern Tasmania by Re–Os black shale geochronology and the ambiguity of "Sturtian"-type diamictite–cap carbonate pairs as chronostratigraphic marker horizons. Precamb. Res. 172, 301–310 (2009).

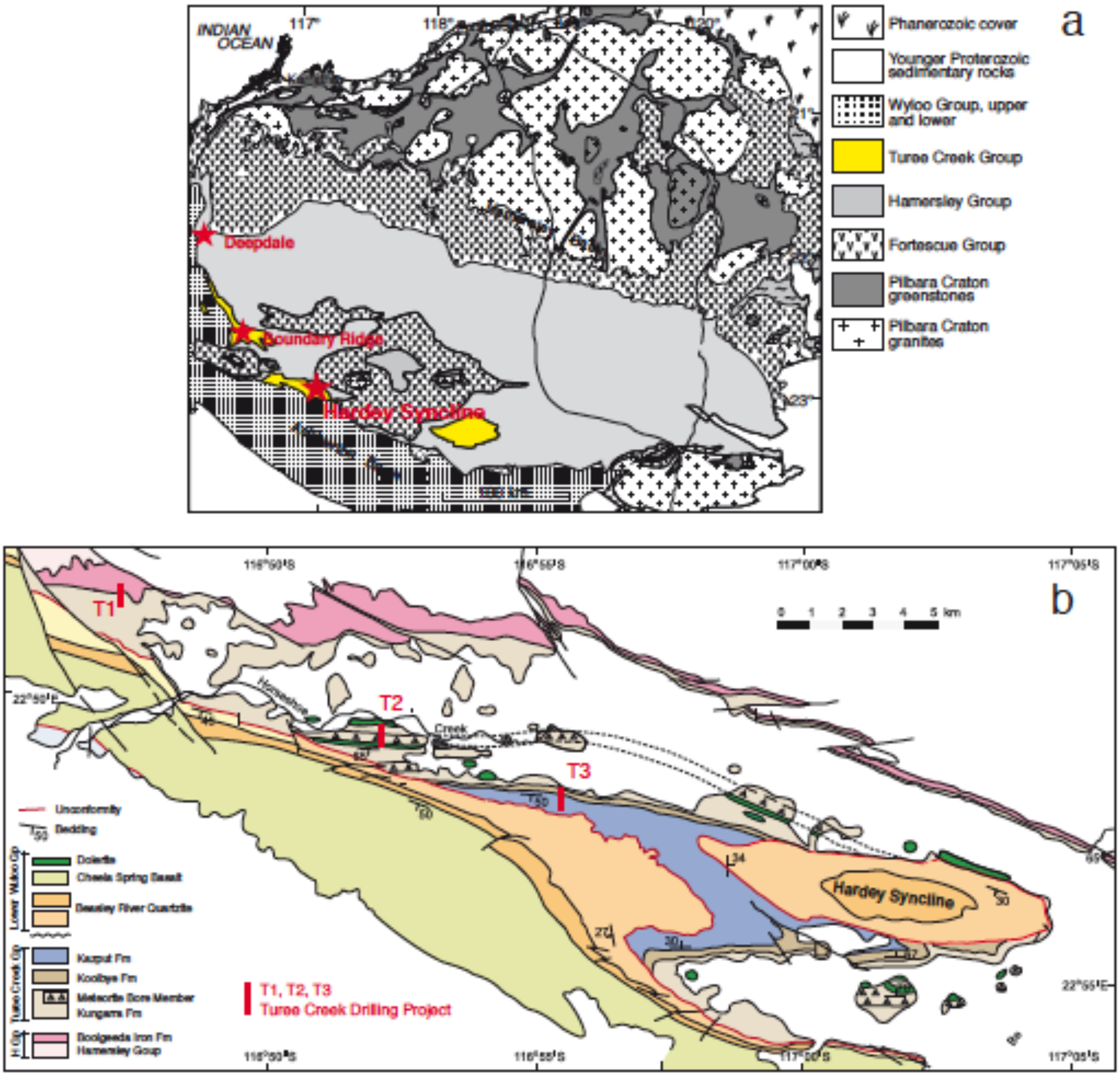

Supplementary Figure 1. a, Geological map of the Turee Creek Group showing the location of the Hardey Syncline, Boundary Ridge and Deepdale locations discussed in the text. **b,** Geological map of the Hardey Syncline modified after Martin et al. (2000)[5] and Van Kranendonk et al., (2015)[6]. The location of the three drill cores T1, T2 and T3 of the Turee Creek Drilling Project are indicated.

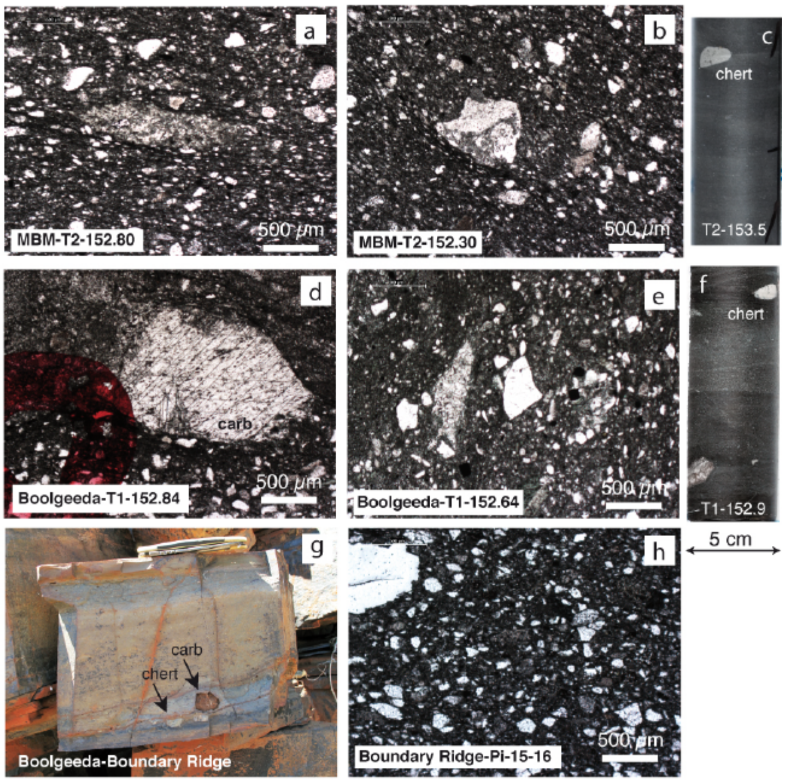

Supplementary Figure 2. Different types of glaciogenic diamictites. a-c, Photomicrographs (**a, b**) and drill core sample (**c,** T2) of the Meteorite Bore Member at the Hardey Syncline locality. **d-f,** Photomicrographs (**c, d**) and drill core sample (**f,** T1) of the diamictite horizon within the Boolgeeda Iron Formation at the Hardey Syncline locality. **g,h,** Surface exposure (**g,** 10 cm-long knife for scale) and photomicrograph of surface sample Pi-15-16 (**h**) of the diamictite horizon in the Boolgeeda Iron Formation at the Boundary Ridge locality. Note the similar texture and occurrence of carbonate (carb) and chert dropstones in the Boolgeeda BIF glaciogenic horizons at Hardey Syncline and Boundary Ridge.

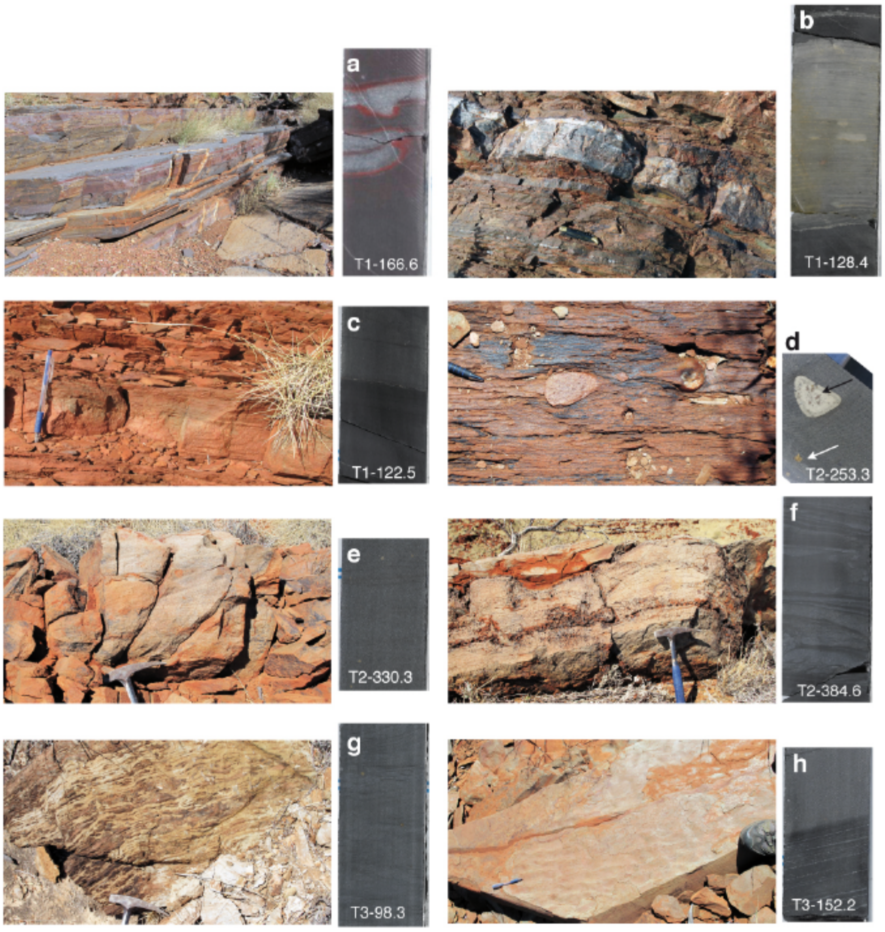

Supplementary Figure 3. Photographs of drill core samples and corresponding surface exposures. Numbers refer to depth of drilling. Cores are 5 cm large. **a**, Iron formation (T1, Boolgeeda Iron Fm). **b**, Chert layer marking the contact between the Boolgeeda IF and overlying Kungarra Fm (T1). **c**, Finely laminated siltstone with cross laminations (T1, Kungarra Fm). **d**, Diamictite of the Meteorite Bore Member (T2). The two arrows point to sulphides in the rock matrix (analysed for their S-isotope composition) and in pebbles (not analysed). **e**, Five to 10 metres thick sand bar located beneath the MBM diamictites (T2, Kungarra Fm). **f**, Carbonate stromatolite located beneath the MBM diamictites (T2, Kungarra Fm). **g**, Carbonate of the Kazput Fm (T3). **h**, Finely laminated siltstone with cross laminations (T3, Kazput Fm.).

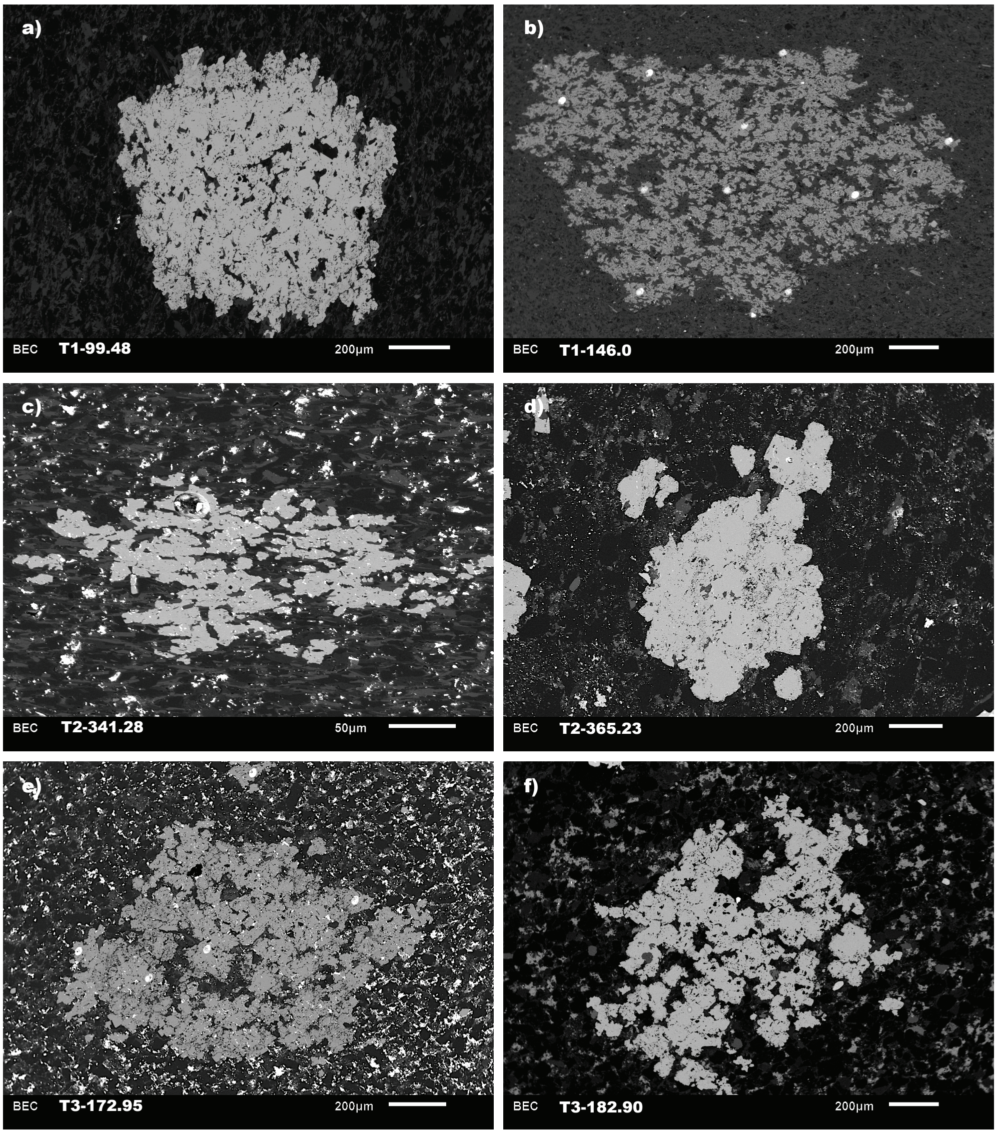

Supplementary Figure 4. **Backscattered electron (BSE) images of syngenetic and diagenetic pyrites analysed in this study.** **a,** inclusion-rich nodular pyrite aggregate (sample T1-99.48), **b,** clusters of microcrystalline pyrites in green mudstone (sample T1-146.0), **c,** clusters of elongated anhedral pyrite crystals typically aligned with bedding observed in mudstone (sample T2-341.28), **d,** compacted inclusion-rich pyrite nodule in laminated mudstone (sample T2-365.23), **e and f,** inclusion-rich nodular aggregates in mudstone (samples T3-172.95 and T3-182.90).

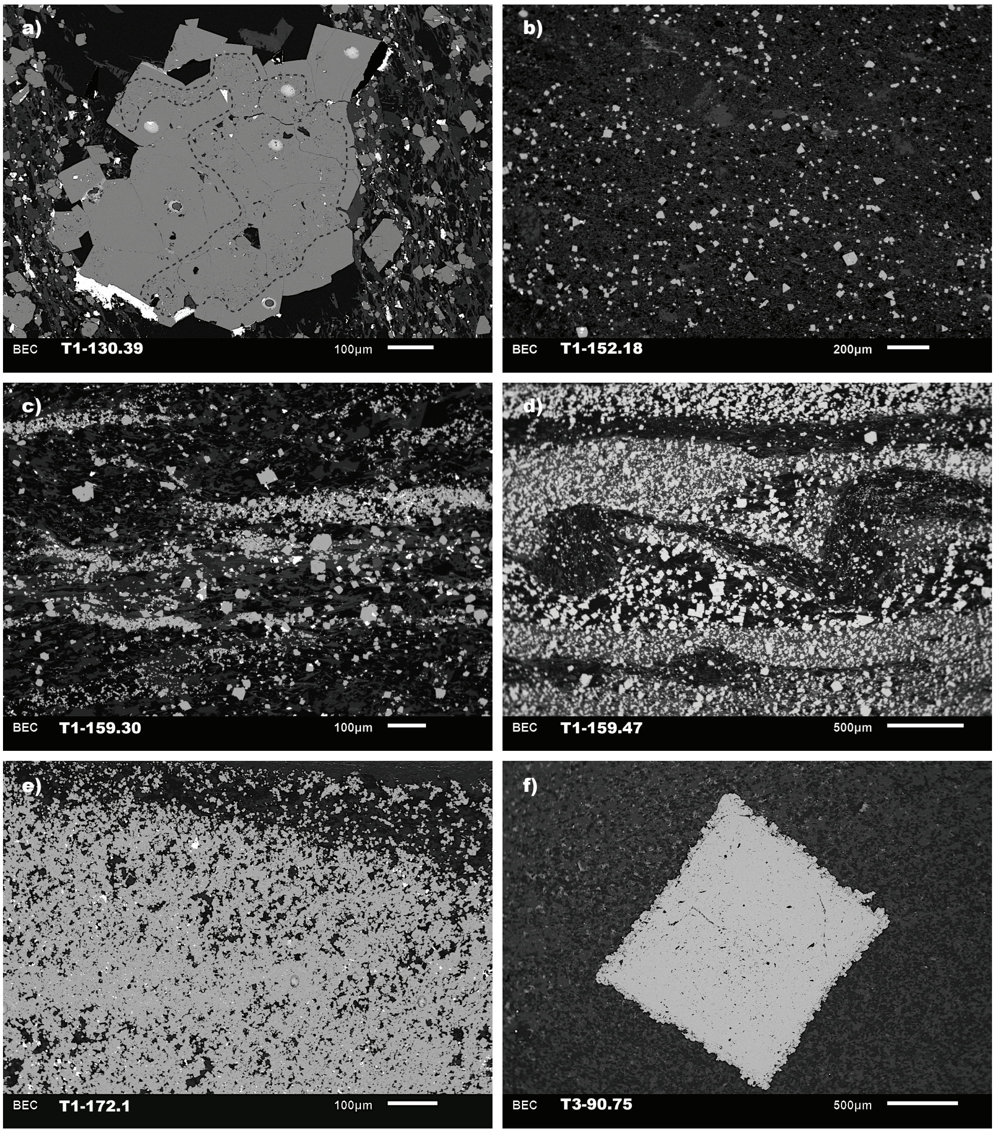

Supplementary Figure 5. Backscattered electron (BSE) images of syngenetic and diagenetic pyrites analysed in this study. a, inclusion-free overgrowth around syngenetic to early diagenetic pyrite framboids observed in green mudstone (sample T1-130.39), **b,** finely disseminated euhedral to subhedral pyrite crystals (sample T1-152.18), **c,** small euhedral to subhedral pyrite crystals (< 25 µm) aligned with bedding in green mudstone (sample T1-159.30), **d,** small euhedral to subhedral pyrite crystals (< 25 µm) conform to soft sediment deformation structures observed in green mudstone (sample T1-159.47), **e,** band of densely packed microcrystalline pyrite aggregates (sample T1-172.1), **f,** anhedral overgrowth around large euhedral pyrite crystal in carbonate (sample T3-90.75).

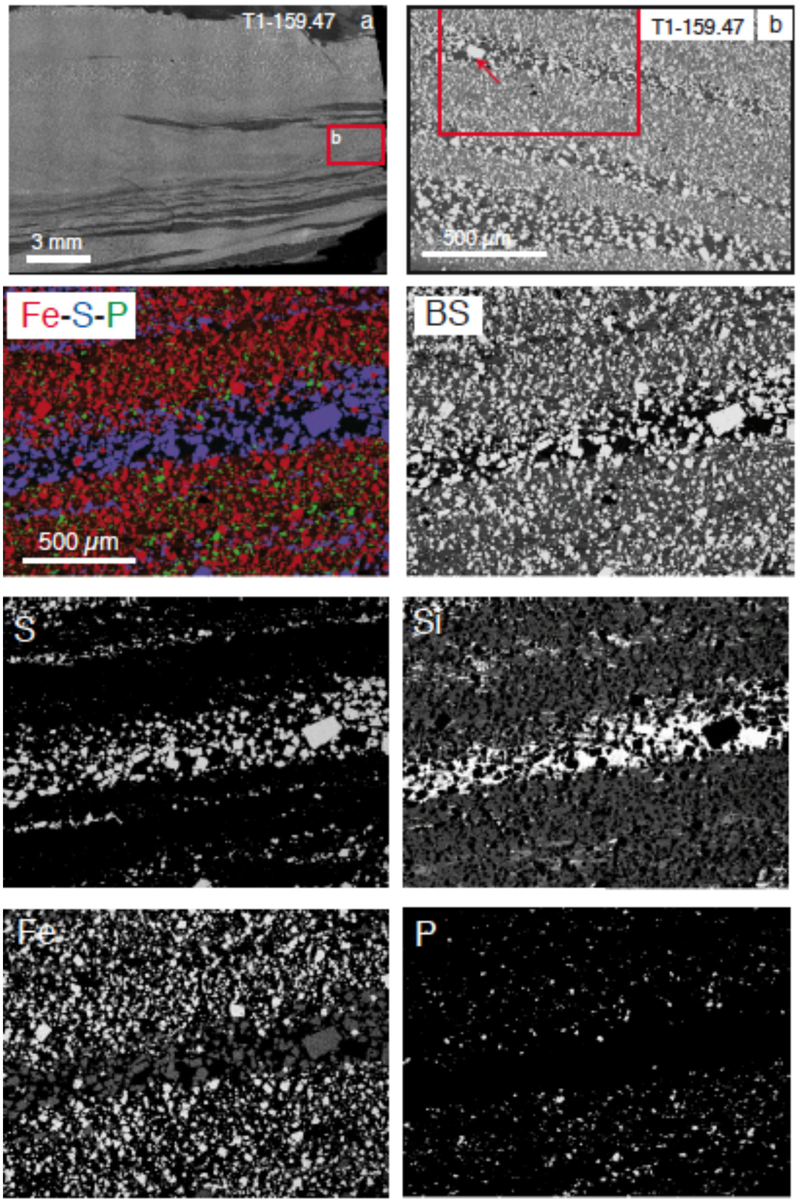

Supplementary Figure 6. X-ray maps of sulphide layers of drill core sample T1-159.47. **a, b,** Photo-micrographs of the petrographic thin section analysed. The red boxes represent zoom in views. Soft sediment deformations are locally preserved in this sample (Supplementary Figure 6d). **BS**, Electron backscattered image, **Fe-S-P**, composite X-ray map showing Fe-oxide (red), pyrite (purple) and apatite (green). Other X-ray maps correspond to sulphur (**S**), silicon (**Si**), iron (**Fe**) and phosphorus (**P**). Note that the sulphide-bearing layers are mainly composed of sulphide and silica.

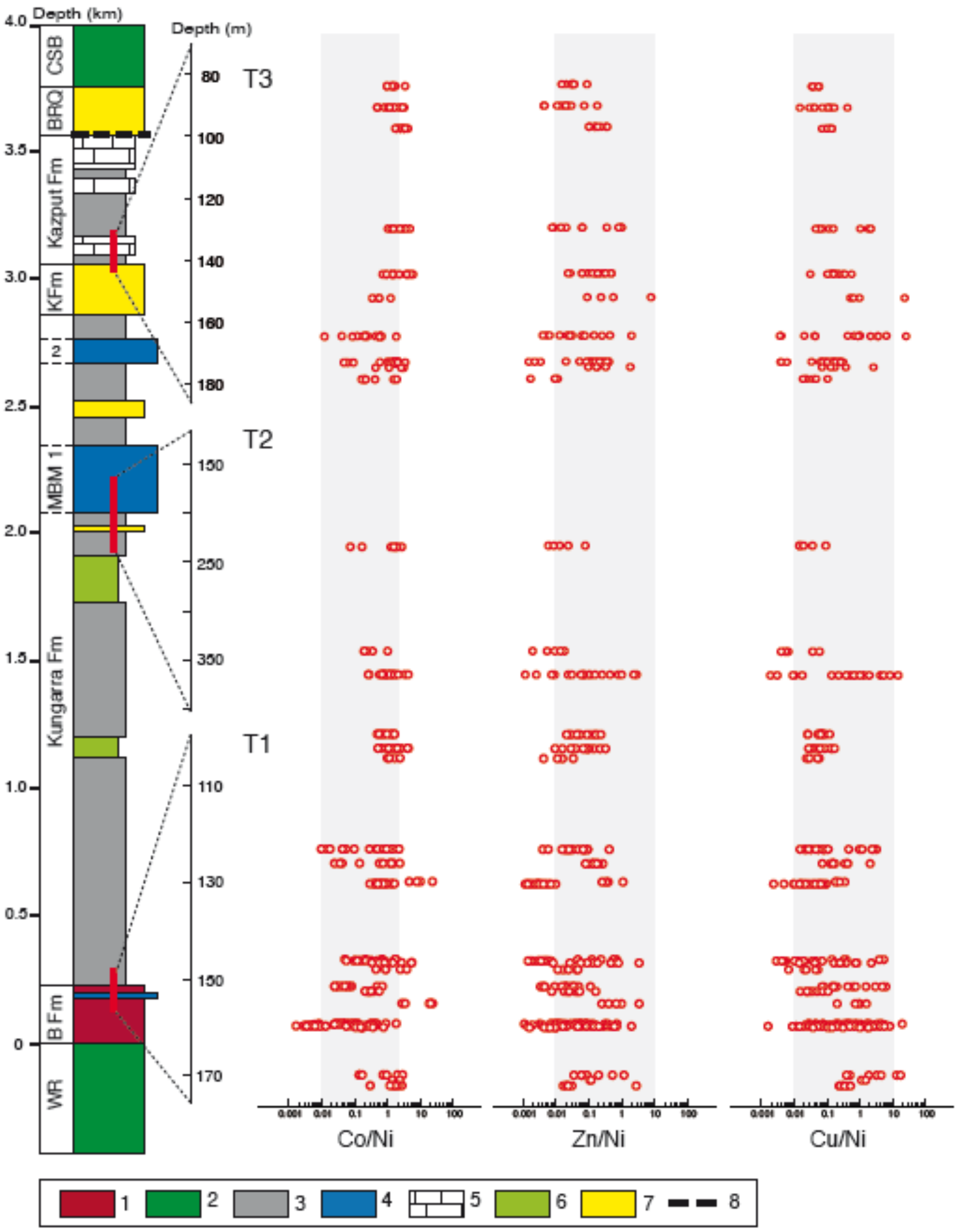

Supplementary Figure 7. Co/Ni, Zn/Ni, and Cu/Ni depth profiles of pyrite from Turee Creek drill cores (T1, T2, and T3). Red circles correspond to individual (spot) analyses carried out with LA-ICP-MS. The grey shaded areas represent the composition limits defined for sedimentary pyrites (0.01 < Co/Ni <2, 0.01 < Cu/Ni < 10, 0.01 < Zn/Ni < 10). Despite the large range observed on Co/Ni, Zn/Ni, and Cu/Ni ratios measured, most of the samples analysed show inter-element ratios within the range of sedimentary pyrite[17,22].

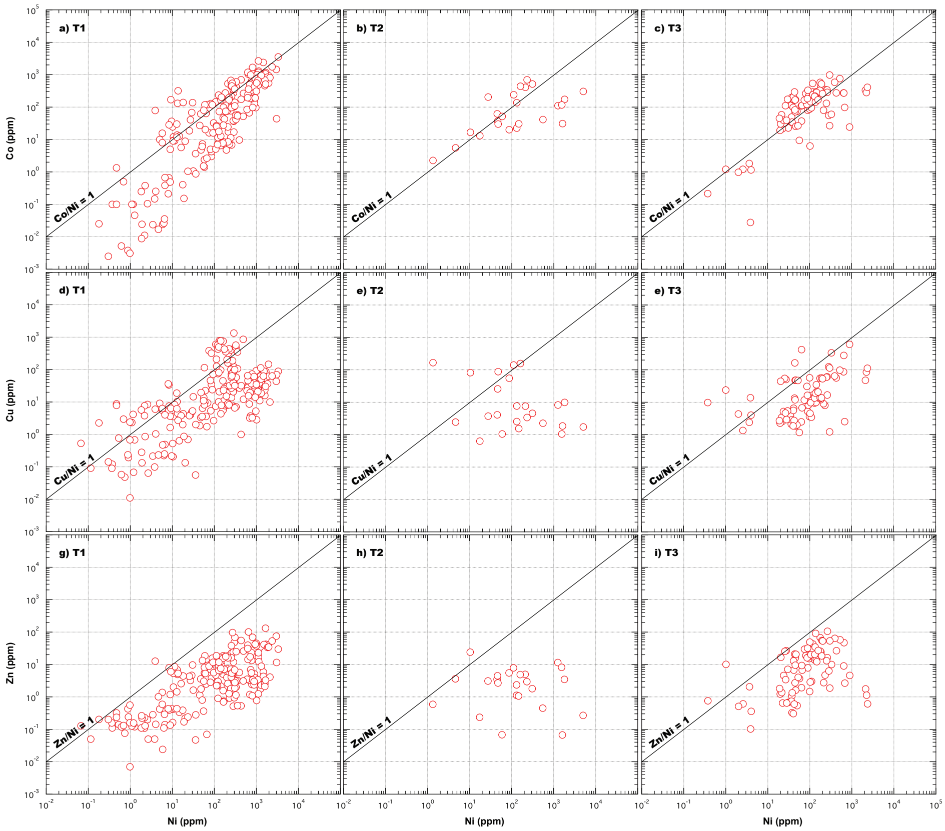

Supplementary Figure 8. Binary plots for Co, Cu, and Zn vs Ni for pyrites from Turee Creek drill cores (T1, T2, and T3). Red circles correspond to individual (spot) analyses carried out with LA-ICP-MS. Cobalt correlates moderately well with Ni, with most of the samples from drill cores T1 (a), T2 (b), and T3 (c) plotting along the Co/Ni = 1 line or on a linear array parallel to Co/Ni =1. A moderate to weak correlation is observed for Cu vs Ni (d, e, f) and Zn vs Ni (g, h, i), probably due to the scatter of the analytical data, which could be related to different trace element incorporation mechanisms into pyrite (i.e., trace element held within the pyrite structure or as nano-inclusions[16,17].

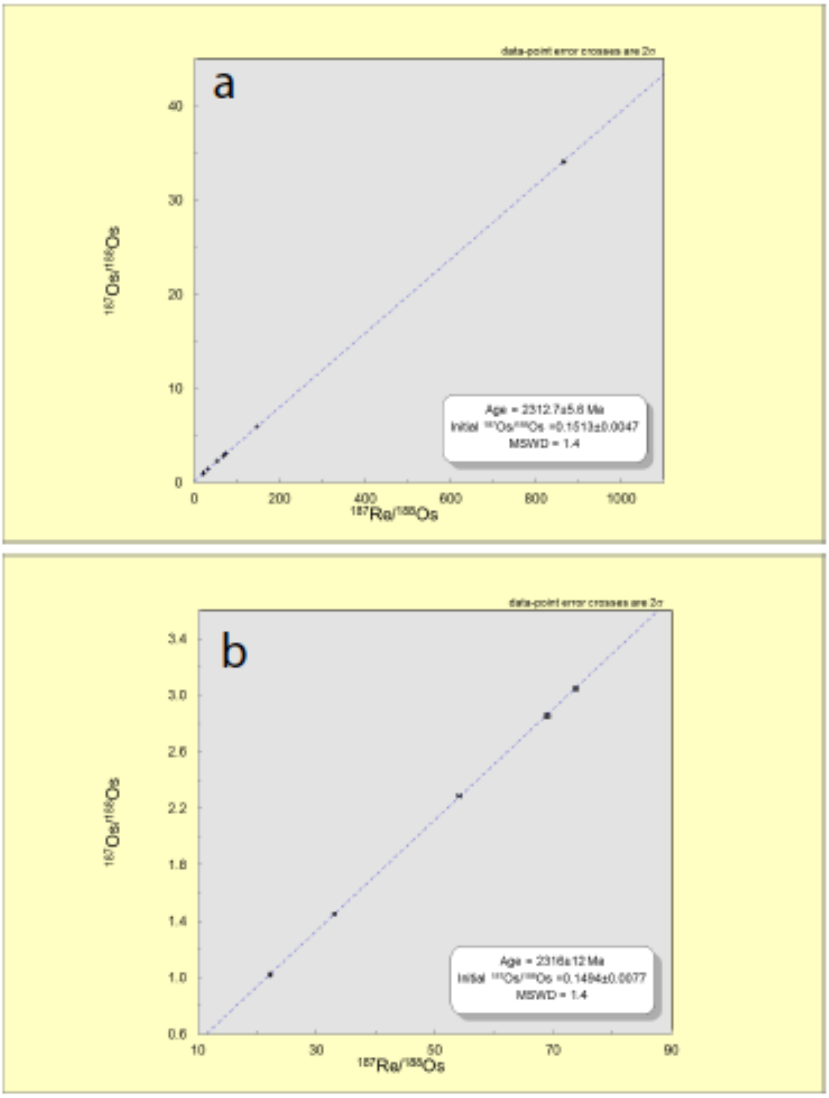

Supplementary Figure 9. **Re-Os age.** Re-Os isochron plots for **(a)** all samples (two pyrite separates and 5 bulk rock samples). **(b)** Plot showing only Re and Os data for diamictite samples collected within a 20 meters depth range using $CrO_3 - H_2SO_4$ digestion medium. Error bars are ± 2 σ. Regression based on Isoplot[12].

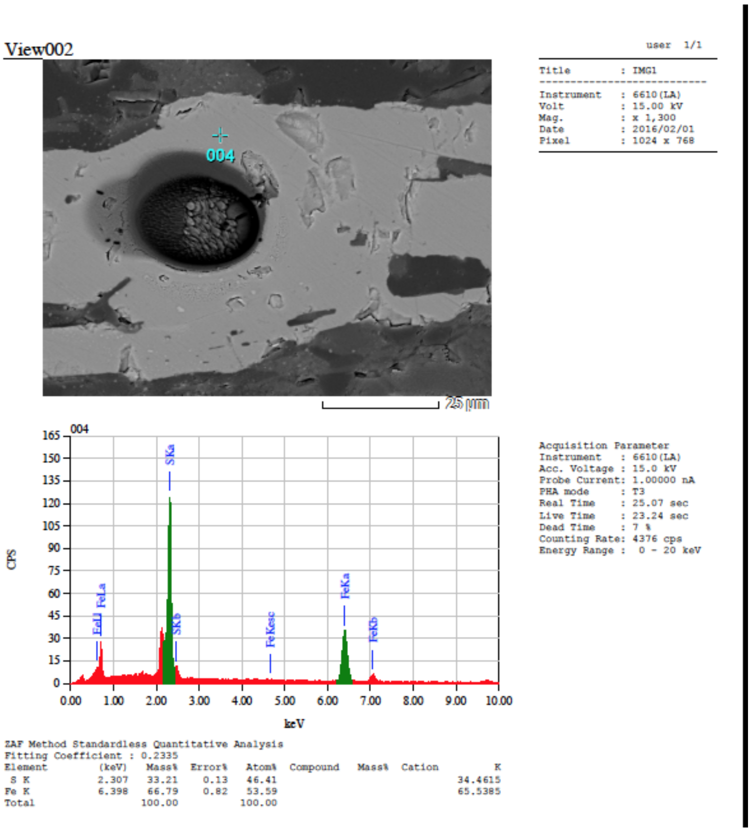

Supplementary Figure 10. Photomicrograph of a sulphide analysed for its S-isotope composition using the SHRIMP SI (ablation crater of the Cs beam) and its major and trace element composition using SEM (analysis 004 showing the two main peaks of S and Fe characteristic of pyrite).

Supplementary Table 1. Re-Os concentrations and Os isotopic compositions for diamictites and pyrite separates.

Sample	Lithology	Re ppb	Total Os ppt	$^{187}Re/^{188}Os$	± 2σ	$^{187}Os/^{188}Os$	± 2σ	Rho*
T2 – 252.55	Diamictite	3.70	350	69.02	0.41	2.855	0.018	0.35
T2 – 259.3	Diamictite	3.68	629	33.03	0.17	1.450	0.002	0.47
T2 – 264.3	Diamictite	4.32	391	73.84	0.41	3.048	0.012	0.53
T2 – 271.1	Diamictite	3.59	870	22.22	0.12	1.022	0.002	0.40
T2 - 272.46	Diamictite	4.05	418	54.20	0.31	2.288	0.005	0.46
T2-272.46	Py	2.89	109	865.93	3.30	34.090	0.010	0.54
T2 - 272.46	Py	3.58	206	147.14	0.52	5.940	0.004	0.57

Rho* stands for error correlation[23].
