# Supplementary material for: Globally asynchronous sulphur isotope signals require re-definition of the Great Oxidation Event
Source: Nat Commun. 2018 Jun 8;9:2245. doi: 10.1038/s41467-018-04621-x (PMC5993798; doi:10.1038/s41467-018-04621-x)
Supplement: Supplementary file 3 — Description of Additional Supplementary Files [file 41467_2018_4621_MOESM3_ESM.pdf]

Description of Additional Supplementary Files

File Name: Supplementary Data 1
Description: LA-ICPMS trace metal abundances of pyrite.

File Name: Supplementary Data 2
Description: Sulphur content.

File Name: Supplementary Data 3
Description: Bulk sulphur isotope analyses.

File Name: Supplementary Data 4
Description: SHRIMP-SI in situ multiple sulphur isotopic compositions (all analyses are pyrite except where indicated).
